# Cullin 3 Ubiquitin Ligases in Cancer Biology: Functions and Therapeutic Implications

**DOI:** 10.3389/fonc.2016.00113

**Published:** 2016-05-02

**Authors:** Hsin-Yi Chen, Ruey-Hwa Chen

**Affiliations:** ^1^Graduate Institute of Cancer Biology and Drug Discovery, College of Medical Science and Technology, Taipei Medical University, Taipei, Taiwan; ^2^Institute of Biological Chemistry, Academia Sinica, Taipei, Taiwan; ^3^Institute of Biochemical Sciences, College of Life Science, National Taiwan University, Taipei, Taiwan

**Keywords:** Cul3 ubiquitin ligases, cancer, Keap1, KLHL20, SPOP

## Abstract

Cullin-RING ubiquitin ligases are the largest E3 ligase family in eukaryotes and are multiprotein complexes. In these complexes, the Cullin protein serves as a scaffold to connect two functional modules of the ligases, the catalytic subunit and substrate-binding subunit. To date, eight members of the Cullin family proteins have been identified. In the Cul3 ubiquitin ligases, Bric-a-brac/Tramtrack/Broad complex (BTB) domain-containing proteins function as a bridge to connect Cul3 and substrates. While the BTB domain is responsible for Cul3 binding, these proteins usually contain an additional domain for substrate interaction, such as MATH, kelch, Zn finger, and PAM, Highwire, and RPM-1 (PHR domain). With the existence of a large number of BTB proteins in human, the Cul3 ubiquitin ligases ubiquitinate a wide range of substrates involving in diverse cellular functions. In this review, we will discuss recent advances on the functions of Cul3 ubiquitin ligases in cancer development, progression, and therapeutic response and the dysregulation of Cul3-mediated ubiquitination events in human malignancies. In particular, we will focus on three Cul3 substrate adaptors, kelch-like ECH-associated protein (Keap1), kelch-like family member 20 (KLHL20), and speckle type BTB/POZ protein (SPOP), with the intent to highlight novel targets in cancer therapy.

## Introduction

The ubiquitin–proteasome system controls a wide range of physiological processes and disease conditions, including cancer. In this system, the addition of ubiquitin moiety to the lysine residue of protein is mediated by a cascade of enzymatic reactions involving E1 activating enzyme, E2 conjugation enzyme, and E3 ubiquitin ligase, in which substrate specificity is conferred by E3 ubiquitin ligase (1, 2). Cullin-RING multiprotein complexes comprise the largest family of ubiquitin ligases, in which one particular Cullin serves as a scaffold for linking two functional modules: the catalytic RING finger protein Roc1 or Roc2 and the substrate-binding module for bringing substrate within the proximity to the catalytic module (3). The human Cullin family consists of eight members: Cul1, Cul2, Cul3, Cul4A, Cul4B, Cul5, Cul7, and Cul9. In the Cul3 family of ubiquitin ligases, the Bric-a-brac/Tramtrack/Broad complex (BTB) domain-containing protein functions as the substrate adaptor to bridge Cul3 and substrate and, therefore, is in analogous to the Skp1–F-box heterodimer in the Cul1 complex (4, 5). Structural analysis indicates that the BTB domain adopts a five α-helical fold resembling other Cullin-binding proteins in the Cullin-RING ligase complexes, such as Skp1 and ElonginC (6). However, two features are unique for the BTB-domain proteins among the substrate adaptors of Cullin family. First, BTB proteins are capable of dimerization and, therefore, can organize two Cul3 molecules in one E3 ligase complex. Second, many BTB proteins contain additional domains and can be classified into subfamilies based on these domains, such as MATH, kelch, Zn finger, and PAM, Highwire, and RPM-1 (PHR). These additional domains are responsible for the interaction of BTB proteins with the substrate of Cul3 complex. Although human genome encodes ~200 BTB proteins, not all of them serve as substrate adaptors of Cul3 ubiquitin ligases. For instance, BTBD12, which lacks a 3-box region critical for binding Cul3, does not copurify with Cul3 from cells (7). In addition, KLHL39, which contains certain non-conserved residues in the BTB domain, fails to bind Cul3 (8).

With the existence of a large number of substrate adaptors, Cul3 ubiquitin ligases have recently been shown to participate in diverse cellular processes, such as cell cycle regulation, protein trafficking, development, and stress responses. In human, functional alterations of this family of ubiquitin ligases are associated with several disease states, such as muscle diseases, metabolic disorders, and cancers (4). This review will provide insights into the functions of Cul3 ligases in tumorigenesis and progression, their dysregulation in human cancers, and therapeutic implications. In particular, we will focus on Cul3 complexes containing the following three substrate adaptors, kelch-like ECH-associated protein (Keap1), kelch-like family member 20 (KLHL20), and speckle type BTB/POZ protein (SPOP). All three proteins bind Cul3 through their BTB domain and elicit profound effects on tumorigenesis and progression.

## The Dual Roles of Keap1–Nrf2 Pathway in Cancer

Kelch-like ECH-associated protein is a Cul3 substrate adaptor containing BACK and kelch-repeat domains in addition to the BTB domain (9–11). Keap1 was first discovered as a key inhibitor of the transcription factor Nf-E2-related factor 2 (Nrf2) (12, 13), which binds to the antioxidant response element (ARE) present in the promoters of downstream genes encoding proteins participating in the cellular antioxidant responses and detoxification of xenobiotics and drugs (14). Under basal conditions, Keap1-based Cul3 complex targets Nrf2 for ubiquitin-dependent degradation (9–11). In the presence of oxidative or electrophilic stress, a number of reactive cysteine residues in Keap1 are covalently modified, leading to its conformational change to prevent Nrf2 ubiquitination. Consequently, Nrf2 is stabilized and undergoes nuclear translocation. Through this mechanism, the Keap1–Nrf2 pathway plays a major role in anti-oxidation and cell defense responses.

Since oxidative stress plays an important role in carcinogenesis, the chemopreventive function of Nrf2 is expected to suppress the initiation of carcinogenesis. In support of this notion, Nrf2^−/−^ mice are more prone to chemical carcinogen-induced tumor formation in the stomach, bladder, and skin (15–19). In addition, Nrf2 deficiency accelerates tumor growth in a mouse lung cancer model induced by B-Raf^V600E^ (20). These findings suggest that activation of Keap1–Nrf2 pathway could be used as a chemopreventive strategy.

Although the chemopreventive function of Keap1–Nrf2 pathway protects normal cells from carcinogenesis, once tumor is formed, cancer cells hijack this pathway for acquiring survival and growth advantage to cope with stressed conditions. For instance, increased Nrf2 expression in cancer cells decreases their sensitivity to a variety of chemotherapeutic agents as well as ionizing radiation, whereas Nrf2 knockdown sensitizes them to cancer therapy (21–24). A similar chemoresistant phenotype is found in cancer cells with elevated Nrf2 activity due to reduced Keap1 expression (21). Although Nrf2-induced activation of antioxidant enzymes accounts for one mechanism of its chemoresistance/radioresistance effect, Nrf2 can also cross talk with other pathways to affect tumor-cell survival. For instance, increased Nrf2 expression is shown to interfere with p53-induced apoptosis (25). Apart from conferring the resistance of tumor cells to therapy, Keap1–Nrf2 pathway also promotes proliferation. It has been found that Nrf2 expression is elevated in response to several oncogenes, such as K-Ras, B-Raf, and Myc. As a consequence, Nrf2-mediated antioxidant responses suppress ROS production in response to the activation of oncogenes, and Nrf2 deficiency suppresses oncogene-induced proliferation and tumorigenesis (26). Evidence has emerged that the proliferative effect of Keap1–Nrf2 pathway is associated with metabolic reprograming (27). Several genes in the pentose phosphate pathway, such as glucose-6-phosphate dehydrogenase (*G6PD*), phosphogluconate dehydrogenase (*PGD*), transaldolase 1 (*TALDO1*), and transketolase (*TKT*), are Nrf2 targets. Nrf2 also activates other metabolic genes, such as malic enzyme 1 (*ME1*), phosphoribosyl pyrophosphate amidotransferase (*PPAT*), methylenetetrahydrofolate dehydrogenase 2 (*MTHFD2*), and isocitrate dehydrogenase 1 (*IDH1*). These enzymes support NADPH generation, purine production, and glucose flux, thereby providing cancer cells with energy and building blocks of DNA and RNA for accelerating proliferation.

The dual functions of Nrf2 in cancer are nicely demonstrated by a mouse model of carcinogenesis. It has been found that Nrf2^−/−^ mice show increased tumor formation at 8th week, after the administration of carcinogen urethane, but become less advanced in malignant progression at 16th week (28). Such finding also suggests that transient activation of Nrf2 in normal cells is protective but prolonged Nrf2 activity enhances tumor survival and progression.

## Dysregulation of Keap1–Nrf2 Pathway in Cancer

Accumulating evidence indicates that Nrf2 expression is aberrantly elevated in many types of cancer. Dysregulation of Nrf2 in tumors can be mediated by multiple mechanisms. Among them, somatic mutations in the components of Keap1–Nrf2 pathway have been found in many cancer types. Loss-of-function mutations in *KEAP1* were initially identified in human lung adenocarcinoma cell lines, which cause reduced affinity of Keap1 to Nrf2 (29). Since then, Keap1 mutations have also been found in several other cancer types, such as NSCLC, gallbladder, ovarian, and liver cancers (21, 30–33). In some cases, Keap1 mutations have a dominant-negative effect on wild-type Keap1, and thus, a heterozygous mutation is sufficient to cause Nrf2 activation (34). As to Nrf2, gain-of-function mutations have been found in several cancers, including lung, head and neck, and esophageal carcinoma (35, 36). The mutations are found exclusively within the DLG and ETGE motifs of Nrf2, which are both required for binding to Keap1 (37). Genetic alterations of *KEAP1* or *NFE2L2* (Nrf2 gene) in cancers, especially in lung cancers, are also uncovered by large-scale omic project (38). In addition to Keap1 and Nrf2, somatic mutations leading to Nrf2 accumulation in cancers have also been discovered in other key components of the Keap1–Nrf2 pathway, such as Cul3 and Rbx1 (39–41).

Kelch-like ECH-associated protein downregulation in cancers can also be mediated by epigenetic mechanisms. For instance, hypermethylation of the *KEAP1* promoter has been found in lung, prostate, malignant glioma, and colorectal cancers, leading to Nrf2 accumulation (42–44). In some cases, *KEAP1* hypermethylation is associated with poor prognosis of patients (45). Besides DNA methylation, miRNA-induced silencing is another mechanism for modulating Keap1–Nrf2 pathway in cancer. For instance, miR-200a, which is frequently repressed in cancer, is found to target Keap1, thereby indirectly regulating Nrf2 (46). In addition, downregulation of several Nrf2 targeting miRNAs in esophageal squamous cell carcinoma is found to associate with poor prognosis (47).

Additional mechanism for regulating Keap1–Nrf2 pathway involves proteins that disrupt the Keap1–Nrf2 interaction. One such protein is p62 (also known as sequestosome 1), which contains an STGE motif that is similar to the ETGE motif in Nrf2. Through this motif, p62 functions as a pseudosubstrate of Keap1 by competing with Nrf2 for Keap1 binding (48–51). Interestingly, p62 is a transcriptional target of Nrf2, indicating the existence of feedback regulation between the two proteins (49). By acting as an autophagic cargo, p62 level is elevated in response to the blockage autophagic flux. In this context, the elevated p62 sequestrates Keap1 in the autophagosome, thereby stabilizing Nrf2 (52). Moreover, p62 phosphorylation through an mTOR-dependent mechanism increases its affinity to Keap1, leading to persistent Nrf2 activation to enhance tumor growth (53). While p62 interrupts Keap1–Nrf2 pathway by binding to Keap1, the p53 downstream target p21 binds to the ETGE and DLG motifs of Nrf2 to prevent its recruitment to Keap1 (54). A similar effect was observed from the tumor suppressor BRCA1, which interacts with the ETGE motif to prevent the binding of Nrf2 to Keap1 (55).

Dysregulation of Keap1–Nrf2 pathway in cancer can also be mediated by metabolites. In the hereditary type 2 papillary renal cell carcinoma, homozygous loss-of-function mutation in the fumarate hydratase leads to the accumulation of fumarate, a metabolite of the Krebs cycle. The excessive fumarate forms adduct on the cysteine residues of Keap1, thereby preventing Nrf2 ubiquitination to promote tumorigenesis (56, 57).

## Targeting Keap1–Nrf2 Pathway for Cancer Prevention and Cancer Treatment

Given the dual roles of Keap1–Nrf2 pathway in cancer, manipulation of this pathway could in principle offer therapeutic benefits. For instance, compounds that activate Nrf2 may be used for cancer prevention, whereas Nrf2 inhibitors could be used as adjuvants in chemotherapy to overcome chemoresistance. Among the Nrf2 activators, many are naturally existing phytochemicals. The prototype and most studied agent is SFN found in cruciferous vegetables. SFN has been shown to exert chemopreventive effect against several cancer types, such as colon, skin, lung, and stomach cancers (58). To date, certain Keap1–Nrf2 activating agents have been tested in clinical trials for their chemopreventive effects against various types of cancer (59, 60). As to Nrf2 inhibitors, a number of small molecules have been identified to inhibit Nrf2 expression or activity, such as IM3829 and brusatol (61, 62). In addition to directly manipulating Nrf2, autophagy pathway that intersects with Keap1–Nrf2 pathway *via* p62-dependent degradation may also be used as a strategy to modulate the activity of Nrf2.

## KLHL20–DAPK Pathway in Interferon Responses

Kelch-like family member 20 possesses the same domain architecture as Keap1, that is, an N-terminal BTB domain, followed by a BACK domain and six kelch repeats. Similar to Keap1 and many other KLHL proteins, KLHL20 binds to Cul3 through its BTB domain to function as a substrate adaptor of Cul3 ubiquitin ligase (63). This protein was uncovered in our laboratory as an interacting partner of death-associated protein kinase (DAPK), a tumor-suppressor protein involved in several cell death paradigms, including apoptosis, autophagic death, and programed necrosis (64–66). In addition to promoting cell death, DAPK elicits other anticancer functions, such as suppressing cell migration and adhesion and promoting cytoskeleton remodeling (67, 68). Consistent with these pleiotropic tumor-suppressive functions, DAPK expression or activity is often suppressed in tumors by epigenetic, posttranscriptional, or posttranslational mechanisms (64, 69–71). The finding that DAPK binds to the kelch-repeat domain of KLHL20 suggests its function as a substrate of the Cul3–KLHL20 ubiquitin ligase. Subsequent biochemical analyses have validated this notion. Moreover, KLHL20-dependent ubiquitination results in the degradation of DAPK by proteasomes. Through this mechanism, KLHL20 antagonizes the cell death-promoting effect of DAPK (63).

Death-associated protein kinase was originally discovered based on its involvement in interferon (IFN)-induced cell death (72). Interestingly, we found that the KLHL20-mediated DAPK ubiquitination and degradation can also be modulated by IFN, in particular, IFN-α and IFN-γ (63). In response to IFN-α/γ treatment, KLHL20 is relocated to a subnuclear domain called PML–nuclear body (PML–NB). This is due to IFN-α/γ-induced transcriptional upregulation of promyelocytic leukemia (PML) (73, 74), the major component of PML–NBs, along with the competition between PML and DAPK for KLHL20 binding (63). As a consequence, DAPK can no longer gain access to KLHL20 and is, therefore, stabilized under IFN-α/γ treated conditions. The stabilization of pro-death DAPK explains its contribution to IFN-induced apoptosis and autophagic death. In certain multiple myeloma cells where IFN-α/γ cannot induce PML and PML–NBs, DAPK is persistently ubiquitinated and degraded by KLHL20. Importantly, this mechanism contributes to the resistance of these multiple myeloma cells to IFN-based therapy. Thus, the KLHL20–DAPK pathway plays a determining role in the efficacy of IFN-based anticancer therapy.

## KLHL20 Promotes the Degradation of Tumor-Suppressor PML

Death-associated protein kinase is not the only tumor-suppressor protein targeted by KLHL20. The finding that PML competes with DAPK for KLHL20 binding suggests its function as a KLHL20 substrate. The *PML* gene was identified at the break point of the t(15:17) chromosome translocation of acute promyelocytic leukemia, which results in the generation of oncogenic PML–RARα fusion protein (75). The PML protein is crucial for the assembly of PML–NBs and elicits pleiotropic antitumor effects, such as suppression of proliferation, angiogenesis, cell migration, and metastasis, and promotion of apoptosis and senescence (76–79). Additionally, PML regulates cancer cell metabolism and suppresses cancer stem cell maintenance (80, 81). Consistent with these tumor-suppressive functions, the expression of PML protein, but not its mRNA, is frequently lost or reduced in a wide range of human malignancies, such as colon, lung, prostate, breast, brain tumors, germ cell tumors, and non-Hodgkin’s lymphoma (82). Evidence has emerged that ubiquitin-mediated proteasomal degradation is a key mechanism for PML degradation in tumors (83–85). The Cul3–KLHL20 complex is one of the ubiquitin ligases that target PML for ubiquitination and proteasomal degradation. However, two consecutive posttranslational modifications are required for PML binding to KLHL20, that is, phosphorylation at S518 by CDK1/2 followed by prolyl isomerization of the pS518–P519 peptide bond by Pin1 (86). This mechanism allows a cell cycle-dependent regulation of PML. Accordingly, PML abundance is gradually declined with the progression of cell cycle (87), correlating with the gradual increase of CDK1/2 activity. Furthermore, since CDK1/2 activity and Pin1 expression are frequently upregulated in tumors, KLHL20-dependent PML ubiquitination and degradation is expected to be enhanced in tumors. Through degradation of PML, KLHL20 is expected to elicit oncogenic roles by blocking PML tumor-suppressive effects. Indeed, KLHL20 confers tumor-promoting functions, such as transformation, migration, and survival, which are dependent on PML downregulation (86).

## KLHL20 in Tumor Hypoxia Responses

The finding that KLHL20 is a transcriptional target of hypoxia-inducible factor-1 (HIF-1) unravels an additional layer of the regulation of KLHL20-mediated PML ubiquitination (86). HIF-1 and its paralog HIF-2 are key molecules to mediate the adaptation of hypoxia by transcriptional activation of a large panel of genes containing “hypoxia responsive element” (HRE) on their promoters (88). This transcriptional program plays crucial roles in many aspects of cancer biology, including immortalization, autocrine growth, metabolic reprograming, invasion, metastasis, angiogenesis, cancer stem cell maintenance, and resistance to chemotherapy and radiotherapy (89). The promoter of *KLHL20* contains two HREs, which are both involved in hypoxia-induced transactivation (86). Due to the induction of KLHL20 by HIF-1, PML ubiquitination and degradation is potentiated under hypoxia conditions. Interestingly, PML is itself a negative regulator of HIF-1 protein translation through a mechanism involving mTOR repression (76). Thus, the HIF-1-induced, KLHL20-mediated PML degradation together with the PML-induced, mTOR-mediated HIF-1α downregulation should constitute a double-negative feedback loop to maximize HIF-1α accumulation in hypoxia. Indeed, evidence has supported the participation of KLHL20/PML pathway in this feedback regulation to lead to a robust induction of both HIF-1α and HIF-2α in response to hypoxia (86). Thus, KLHL20-mediated PML ubiquitination results in not only the inhibition of PML tumor-suppressive functions but also a robust induction of various tumor hypoxia responses to contribute to the aggressiveness of diseases.

## Dysregulation of KLHL20 in Cancer

Since HIF-1α is frequently upregulated in tumors through hypoxia-dependent or -independent mechanism (90), KLHL20 expression is expected to be upregulated in certain cancers. In line with this notion, KLHL20 expression is elevated in prostate cancers compared to its expression in benign prostatic hyperplasia. Furthermore, this upregulation correlates with dysregulation of several other key molecules in the KLHL20–PML pathway, including HIF-1α upregulation, Pin1 upregulation, and PML downregulation (86). More importantly, patients displaying the signature of high HIF-1α, high KLHL20, high Pin1, and low PML expression pattern are found to be progressively increased with disease progression. These clinical findings support the significance of KLHL20–PML pathway in the progression of prostate cancer and suggest a promise for targeting this pathway in the treatment of aggressive prostate cancers.

In addition to the regulation of its expression level, the activity of Cul3–KLHL20 E3 ligase can be regulated in tumors by an inhibitor (8). Interestingly, this inhibitor, called KLHL39, shares a similar domain structure with KLHL20. However, due to the presence of certain atypical residues in its BTB domain, KLHL39 fails to bind Cul3. Rather, it interacts with KLHL20 through the kelch domain of two proteins. We found that KLHL39 cannot serve as a substrate of the Cul3–KLHL20 ubiquitin ligase but disrupts the interaction of KLHL20 with its substrate such as PML and DAPK. Surprisingly, KLHL39 also blocks the binding of KLHL20 to Cul3 through an unknown mechanism. Through these dual inhibitory roles, i.e., inhibition of KLHL20 binding to Cul3 and substrates, KLHL39 blocks KLHL20-dependent ubiquitination and degradation of DAPK and PML, leading to an increase of their steady-state levels. Clinically, low expression of KLHL39 in human colon cancer correlates with low expression of PML and DAPK, higher tumor grade, lymph node metastasis, and distant metastasis. Furthermore, by comparing the primary tumors with lymph node metastases of the same patient, low expression of each KLHL39, DAPK, and PML is more frequently observed in the metastatic lesions. Consistent with the clinical observations, KLHL39 suppresses colon cancer migration, invasion, and metastasis, and these tumor-suppressive effects are all mediated through a PML- and DAPK-dependent manner. These findings indicate a tumor-suppressive function of KLHL39 by blocking KLHL20-dependent ubiquitination of PML and DAPK.

## The Tumor-Suppressive Functions of SPOP

Speckle type BTB/POZ protein comprises an N-terminal MATH domain, a BTB domain, a 3-box domain, and a C-terminal nuclear localization sequence. Similar to other BTB proteins, SPOP serves as a substrate adaptor of Cul3 ubiquitin ligase, and substrate binding is mediated by its MATH domain, which binds to a SPOP-binding consensus (SBC) motif φ-π-S-S/T-S/T (φ = non-polar; π = polar) on the substrate (7). The linkage of SPOP to cancer was first revealed by cancer genomic analyses, which uncovers SPOP as a significantly mutated gene in human prostate cancers (91). Subsequent analyses using larger prostate cancer patient cohorts confirmed this finding (92–95). Most of these SPOP mutations occur in the MATH domain, suggesting that mutations impair substrate binding. To date, a number of SPOP substrates have been identified in the context of prostate cancer, including androgen receptor (AR), steroid receptor coactivator (SRC)-3, DEK, ERG, and SENP7 (96–101).

Androgen receptor signaling is crucial for prostate cancer initiation, progression, and development of resistance to antiandrogen therapy (102). AR is found as a *bona fide* substrate of SPOP-based Cul3 ubiquitin ligase and an SBC motif in the hinge region of AR mediates its interaction with SPOP (97). SPOP-mediated AR ubiquitination leads to its proteolysis in the proteasome. Importantly, prostate cancer-associated SPOP mutants fail to target AR for ubiquitination, whereas AR splicing mutants lacking hinge domain are refractory to SPOP-mediated degradation. This study also revealed that SPOP-mediated AR degradation is antagonized by androgens and promoted by antiandrogens, suggesting that ligand binding-induced conformational change of AR could affect its recruitment to SPOP. In addition to AR, SPOP binds to SRC-3, a preferred coactivator of hormone-activated AR (103, 104), and targets it for Cul3-mediated ubiquitination and degradation (99). Again, prostate cancer-associated SPOP mutants cannot target SRC-3 for degradation (105). Thus, these SPOP mutants could enhance AR functions in prostate cancers by inhibiting the turnover of both AR and its coactivator SRC-3.

The role of AR in prostate cancer initiation is mediated in part by the translocation of oncogenic ETS family transcription factors, such as ERG and ETV1, to the loci of androgen regulated genes including TMPRSS2 (106, 107). Among them, the most common fusion is TMPRSS2-ERG, which occurs in >50% of prostate cancers. This fusion allows AR-induced ERG overexpression, which elicits oncogenic functions such as proliferation, migration, and invasion (108). Recent studies indicate that ERG is targeted to SPOP-based Cul3 ubiquitin ligase for ubiquitination and degradation, and an SBC motif in the N-terminus of ERG is responsible for SPOP recognition (96, 98). Importantly, prostate cancer-associated SPOP mutants fail to induce ERG degradation, whereas the majority of TMPRSS2-ERG fusions encoding N-terminal truncated ERG proteins are resistant to SPOP-mediated degradation. Since these two types of genetic alterations, i.e., SPOP mutations and TMPRSS2-ERG fusions, similarly lead to ERG stabilization, it is conceivable that their incidences are mutually exclusive in prostate cancers (93).

Using mass spectrometry-based ubiquitylome analysis, several SPOP substrates have been discovered from prostate cancer cells, such as DEK, TRIM24, and NCOA3 (100). Among them, DEK stabilization contributes to prostate cancer invasion and stem cell-like property and DEK upregulation correlates with SPOP mutations, in prostate cancer. Besides these substrates, SENP7 desumoylase is also identified as a SPOP substrate (101). The SPOP–SENP7 axis promotes prostate cancer senescence, which is impaired by the presence of prostate cancer-associated SPOP mutants. Collectively, SPOP targets the degradation of multiple tumor-promoting proteins in prostate cancer to contribute to the carcinogenesis process.

Of note, SPOP mutations in the MATH domain are also found in endometrial cancers (109). In this cancer type, wild-type SPOP, but not cancer-associated SPOP mutants, targets estrogen receptor-α for ubiquitination and degradation (110). In breast cancer, SPOP represents one of the highest loci for loss of heterozygosity (99). Progesterone receptor, which contributes to the development of breast cancer, is found to function as a substrate of SPOP is this cancer type (111). These findings suggest that SPOP governs the turnover of distinct hormone receptors to participate in the carcinogenesis of several cancer types.

## The Tumor-Promoting Functions of SPOP

In contrast to the aforementioned cancer types, SPOP plays a tumor-promoting role in kidney cancer. SPOP high expression occurs in 99% of clear cell renal cell carcinoma (ccRCC) (112), the most prevalent type of kidney cancer. The pathology of ccRCC is tightly associated with HIF-1 accumulation resulted from deficiency of VHL, which acts as a substrate adaptor of Cul2 ubiquitin ligase (113). Importantly, SPOP is a transcriptional target of HIF-1 and hypoxia potentiates the cytoplasmic accumulation of SPOP (114). This cytoplasmic retention of SPOP confers tumor-promoting activities, which is opposite to the function of SPOP in the nucleus. Mechanistically, SPOP controls the ubiquitination and degradation of several tumor suppressors residing in the cytoplasm, such as PTEN, ERK phosphatases, Daxx, and Gli2. In addition to kidney cancer, SPOP is reported to mediate ubiquitination and destabilization of breast cancer metastasis suppressor 1 (BRMS1) in breast cancer, thereby derepressing metastasis-associated genes (115). Thus, SPOP elicits context-dependent functions in cancer development, which is influenced in part by its different subcellular distributions.

## Concluding Remarks

**Figure 1:**
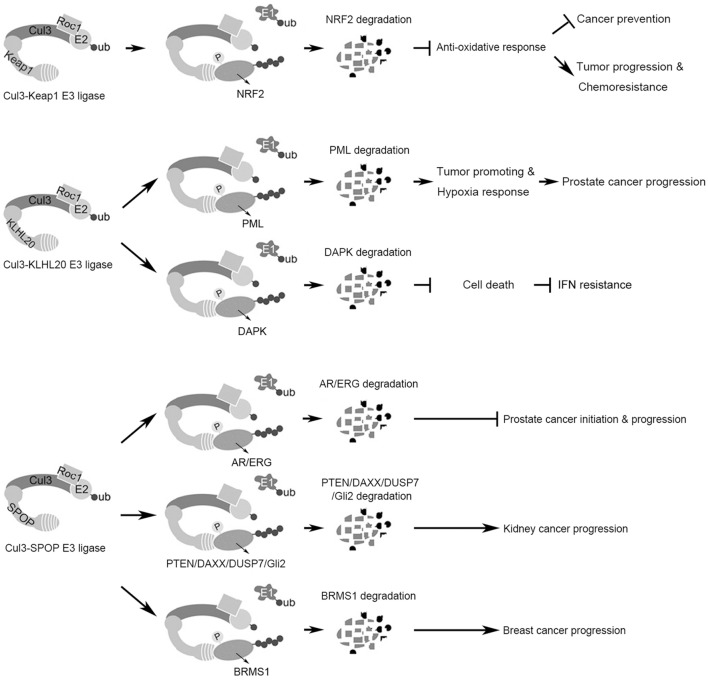
**Summary of the functions and substrates of three Cul3 complexes in cancer**. (Top) The Cul3–Keap1 ubiquitin ligase mediates Nrf2 degradation to suppress antioxidant responses, which plays dual roles in cancer initiation and progression. (Middle) The Cul3–KLHL20 ubiquitin ligase mediates the degradation of tumor-suppressor proteins PML and DAPK, thereby promoting tumor progression and therapy resistance. (Bottom) The Cul3–SPOP ubiquitin ligase possesses context-dependent tumor-promoting or -inhibiting role by regulating the degradation of multiple substrates.

A significant number of recent studies shed light on the biological functions of Cul3 E3 ligases that regulate tumor development, progression, and therapeutic response. In particular, Keap1, KLHL20, and SPOP are the most reported Cul3 substrate adaptors for their impacts on various cancer types. These three proteins mediate Cul3-dependent ubiquitination on multiple substrates to influence on tumor initiation, progression, and therapeutic response (Figure 1). While KLHL20 mainly plays a tumor-promoting role, SPOP elicits both tumor-promoting and suppressive effects depending on its subcellular localization and cell context. As to Keap1, its role in cancer varies with the stages in the multistep carcinogenesis process. Further insights into the functional and mechanistic basis of KLHL20 and SPOP in cancer development can be obtained from studies with suitable animal models, especially genetically engineered mouse models. In this regard, KLHL20 conditional knockout mice are recently generated (116), which could offer a powerful tool for studying its function in multistep carcinogenesis in a tissue-specific manner. In addition, systematic identification and characterization of substrates of these three adaptors and other Cul3 substrate adaptors can potentially facilitate a more comprehensive understanding on the functions of Cul3 ubiquitin ligases in cancer biology and their clinical implications. Such information will be helpful for designing new therapeutic strategies for cancer intervention.

## Author Contributions

Both the authors collected and reviewed literatures and wrote the manuscript.

## Conflict of Interest Statement

The authors declare that the research was conducted in the absence of any commercial or financial relationships that could be construed as a potential conflict of interest.

## References

[1] Ciechanover A Orian A Schwartz AL. Ubiquitin-mediated proteolysis: biological regulation *via* destruction. Bioessays (2000) 22:442–51. 10.1002/(SICI)1521-1878(200005)22:5<442::AID-BIES6>3.0.CO;2-Q 10797484

[2] Hochstrasser M. Ubiquitin-dependent protein degradation. Annu Rev Genet (1996) 30:405–39. 10.1146/annurev.genet.30.1.405 8982460

[3] Petroski MD Deshaies RJ. Function and regulation of cullin-RING ubiquitin ligases. Nat Rev Mol Cell Biol (2005) 6:9–20. 10.1038/nrm1547 15688063

[4] Genschik P Sumara I Lechner E. The emerging family of CULLIN3-RING ubiquitin ligases (CRL3s): cellular functions and disease implications. EMBO J (2013) 32:2307–20. 10.1038/emboj.2013.173 23912815 PMC3770339

[5] Pintard L Willems A Peter M. Cullin-based ubiquitin ligases: Cul3-BTB complexes join the family. EMBO J (2004) 23:1681–7. 10.1038/sj.emboj.7600186 15071497 PMC394240

[6] Stogios PJ Downs GS Jauhal JJ Nandra SK Prive GG. Sequence and structural analysis of BTB domain proteins. Genome Biol (2005) 6:R82. 10.1186/gb-2005-6-10-r82 16207353 PMC1257465

[7] Zhuang M Calabrese MF Liu J Waddell MB Nourse A Hammel M Structures of SPOP-substrate complexes: insights into molecular architectures of BTB-Cul3 ubiquitin ligases. Mol Cell (2009) 36:39–50. 10.1016/j.molcel.2009.09.022 19818708 PMC2847577

[8] Chen HY Hu JY Chen TH Lin YC Liu X Lin MY KLHL39 suppresses colon cancer metastasis by blocking KLHL20-mediated PML and DAPK ubiquitination. Oncogene (2015) 34:5141–51. 10.1038/onc.2014.435 25619834

[9] Cullinan SB Gordan JD Jin J Harper JW Diehl JA. The Keap1-BTB protein is an adaptor that bridges Nrf2 to a Cul3-based E3 ligase: oxidative stress sensing by a Cul3-Keap1 ligase. Mol Cell Biol (2004) 24:8477–86. 10.1128/MCB.24.19.8477-8486.2004 15367669 PMC516753

[10] Kobayashi A Kang MI Okawa H Ohtsuji M Zenke Y Chiba T Oxidative stress sensor Keap1 functions as an adaptor for Cul3-based E3 ligase to regulate proteasomal degradation of Nrf2. Mol Cell Biol (2004) 24:7130–9. 10.1128/MCB.24.16.7130-7139.2004 15282312 PMC479737

[11] Zhang DD Lo SC Cross JV Templeton DJ Hannink M. Keap1 is a redox-regulated substrate adaptor protein for a Cul3-dependent ubiquitin ligase complex. Mol Cell Biol (2004) 24:10941–53. 10.1128/MCB.24.24.10941-10953.2004 15572695 PMC533977

[12] Dinkova-Kostova AT Holtzclaw WD Cole RN Itoh K Wakabayashi N Katoh Y Direct evidence that sulfhydryl groups of Keap1 are the sensors regulating induction of phase 2 enzymes that protect against carcinogens and oxidants. Proc Natl Acad Sci U S A (2002) 99:11908–13. 10.1073/pnas.172398899 12193649 PMC129367

[13] Itoh K Wakabayashi N Katoh Y Ishii T Igarashi K Engel JD Keap1 represses nuclear activation of antioxidant responsive elements by Nrf2 through binding to the amino-terminal Neh2 domain. Genes Dev (1999) 13:76–86. 10.1101/gad.13.1.76 9887101 PMC316370

[14] Kensler TW Wakabayashi N Biswal S. Cell survival responses to environmental stresses *via* the Keap1-Nrf2-ARE pathway. Annu Rev Pharmacol Toxicol (2007) 47:89–116. 10.1146/annurev.pharmtox.46.120604.141046 16968214

[15] Fahey JW Haristoy X Dolan PM Kensler TW Scholtus I Stephenson KK Sulforaphane inhibits extracellular, intracellular, and antibiotic-resistant strains of *Helicobacter pylori* and prevents benzo[a]pyrene-induced stomach tumors. Proc Natl Acad Sci U S A (2002) 99:7610–5. 10.1073/pnas.112203099 12032331 PMC124299

[16] Khor TO Huang MT Prawan A Liu Y Hao X Yu S Increased susceptibility of Nrf2 knockout mice to colitis-associated colorectal cancer. Cancer Prev Res (Phila) (2008) 1:187–91. 10.1158/1940-6207.CAPR-08-0028 19138955 PMC3580177

[17] Osburn WO Karim B Dolan PM Liu G Yamamoto M Huso DL Increased colonic inflammatory injury and formation of aberrant crypt foci in Nrf2-deficient mice upon dextran sulfate treatment. Int J Cancer (2007) 121:1883–91. 10.1002/ijc.22943 17631644

[18] Ramos-Gomez M Kwak MK Dolan PM Itoh K Yamamoto M Talalay P Sensitivity to carcinogenesis is increased and chemoprotective efficacy of enzyme inducers is lost in nrf2 transcription factor-deficient mice. Proc Natl Acad Sci U S A (2001) 98:3410–5. 10.1073/pnas.051618798 11248092 PMC30667

[19] Xu C Huang MT Shen G Yuan X Lin W Khor TO Inhibition of 7,12-dimethylbenz(a)anthracene-induced skin tumorigenesis in C57BL/6 mice by sulforaphane is mediated by nuclear factor E2-related factor 2. Cancer Res (2006) 66:8293–6. 10.1158/0008-5472.CAN-06-0300 16912211

[20] Strohecker AM Guo JY Karsli-Uzunbas G Price SM Chen GJ Mathew R Autophagy sustains mitochondrial glutamine metabolism and growth of BrafV600E-driven lung tumors. Cancer Discov (2013) 3:1272–85. 10.1158/2159-8290.CD-13-0397 23965987 PMC3823822

[21] Shibata T Kokubu A Gotoh M Ojima H Ohta T Yamamoto M Genetic alteration of Keap1 confers constitutive Nrf2 activation and resistance to chemotherapy in gallbladder cancer. Gastroenterology (2008) 135:1358–68. 10.1053/j.gastro.2008.06.082 18692501

[22] Solis LM Behrens C Dong W Suraokar M Ozburn NC Moran CA Nrf2 and Keap1 abnormalities in non-small cell lung carcinoma and association with clinicopathologic features. Clin Cancer Res (2010) 16:3743–53. 10.1158/1078-0432.CCR-09-3352 20534738 PMC2920733

[23] Wang XJ Sun Z Villeneuve NF Zhang S Zhao F Li Y Nrf2 enhances resistance of cancer cells to chemotherapeutic drugs, the dark side of Nrf2. Carcinogenesis (2008) 29:1235–43. 10.1093/carcin/bgn095 18413364 PMC3312612

[24] Zhang P Singh A Yegnasubramanian S Esopi D Kombairaju P Bodas M Loss of Kelch-like ECH-associated protein 1 function in prostate cancer cells causes chemoresistance and radioresistance and promotes tumor growth. Mol Cancer Ther (2010) 9:336–46. 10.1158/1535-7163.MCT-09-0589 20124447 PMC2821808

[25] Faraonio R Vergara P Di Marzo D Pierantoni MG Napolitano M Russo T p53 suppresses the Nrf2-dependent transcription of antioxidant response genes. J Biol Chem (2006) 281:39776–84. 10.1074/jbc.M605707200 17077087

[26] DeNicola GM Karreth FA Humpton TJ Gopinathan A Wei C Frese K Oncogene-induced Nrf2 transcription promotes ROS detoxification and tumorigenesis. Nature (2011) 475:106–9. 10.1038/nature10189 21734707 PMC3404470

[27] Mitsuishi Y Taguchi K Kawatani Y Shibata T Nukiwa T Aburatani H Nrf2 redirects glucose and glutamine into anabolic pathways in metabolic reprogramming. Cancer Cell (2012) 22:66–79. 10.1016/j.ccr.2012.05.016 22789539

[28] Satoh H Moriguchi T Takai J Ebina M Yamamoto M. Nrf2 prevents initiation but accelerates progression through the Kras signaling pathway during lung carcinogenesis. Cancer Res (2013) 73:4158–68. 10.1158/0008-5472.CAN-12-4499 23610445

[29] Padmanabhan B Tong KI Ohta T Nakamura Y Scharlock M Ohtsuji M Structural basis for defects of Keap1 activity provoked by its point mutations in lung cancer. Mol Cell (2006) 21:689–700. 10.1016/j.molcel.2006.01.013 16507366

[30] Konstantinopoulos PA Spentzos D Fountzilas E Francoeur N Sanisetty S Grammatikos AP Keap1 mutations and Nrf2 pathway activation in epithelial ovarian cancer. Cancer Res (2011) 71:5081–9. 10.1158/0008-5472.CAN-10-4668 21676886

[31] Ohta T Iijima K Miyamoto M Nakahara I Tanaka H Ohtsuji M Loss of Keap1 function activates Nrf2 and provides advantages for lung cancer cell growth. Cancer Res (2008) 68:1303–9. 10.1158/0008-5472.CAN-07-5003 18316592

[32] Singh A Misra V Thimmulappa RK Lee H Ames S Hoque MO Dysfunctional KEAP1-NRF2 interaction in non-small-cell lung cancer. PLoS Med (2006) 3:e420. 10.1371/journal.pmed.0030420 17020408 PMC1584412

[33] Yoo NJ Kim HR Kim YR An CH Lee SH. Somatic mutations of the KEAP1 gene in common solid cancers. Histopathology (2012) 60:943–52. 10.1111/j.1365-2559.2012.04178.x 22348534

[34] Suzuki T Maher J Yamamoto M. Select heterozygous Keap1 mutations have a dominant-negative effect on wild-type Keap1 *in vivo*. Cancer Res (2011) 71:1700–9. 10.1158/0008-5472.CAN-10-2939 21177379

[35] Shibata T Kokubu A Saito S Narisawa-Saito M Sasaki H Aoyagi K NRF2 mutation confers malignant potential and resistance to chemoradiation therapy in advanced esophageal squamous cancer. Neoplasia (2011) 13:864–73. 10.1593/neo.11750 21969819 PMC3182278

[36] Shibata T Ohta T Tong KI Kokubu A Odogawa R Tsuta K Cancer related mutations in NRF2 impair its recognition by Keap1-Cul3 E3 ligase and promote malignancy. Proc Natl Acad Sci U S A (2008) 105:13568–73. 10.1073/pnas.0806268105 18757741 PMC2533230

[37] Tong KI Kobayashi A Katsuoka F Yamamoto M. Two-site substrate recognition model for the Keap1-Nrf2 system: a hinge and latch mechanism. Biol Chem (2006) 387:1311–20. 10.1515/BC.2006.164 17081101

[38] Kandoth C McLellan MD Vandin F Ye K Niu B Lu C Mutational landscape and significance across 12 major cancer types. Nature (2013) 502:333–9. 10.1038/nature12634 24132290 PMC3927368

[39] Martinez VD Vucic EA Thu KL Pikor LA Hubaux R Lam WL. Unique pattern of component gene disruption in the NRF2 inhibitor KEAP1/CUL3/RBX1 E3-ubiquitin ligase complex in serous ovarian cancer. Biomed Res Int (2014) 2014:159459. 10.1155/2014/159459 25114896 PMC4121105

[40] Martinez VD Vucic EA Thu KL Pikor LA Lam S Lam WL. Disruption of KEAP1/CUL3/RBX1 E3-ubiquitin ligase complex components by multiple genetic mechanisms: association with poor prognosis in head and neck cancer. Head Neck (2015) 37:727–34. 10.1002/hed.23663 24596130

[41] Ooi A Dykema K Ansari A Petillo D Snider J Kahnoski R CUL3 and NRF2 mutations confer an NRF2 activation phenotype in a sporadic form of papillary renal cell carcinoma. Cancer Res (2013) 73:2044–51. 10.1158/0008-5472.CAN-12-3227 23365135

[42] Hanada N Takahata T Zhou Q Ye X Sun R Itoh J Methylation of the KEAP1 gene promoter region in human colorectal cancer. BMC Cancer (2012) 12:66. 10.1186/1471-2407-12-66 22325485 PMC3296656

[43] Kang KA Piao MJ Kim KC Kang HK Chang WY Park IC Epigenetic modification of Nrf2 in 5-fluorouracil-resistant colon cancer cells: involvement of TET-dependent DNA demethylation. Cell Death Dis (2014) 5:e1183. 10.1038/cddis.2014.149 24743738 PMC4001304

[44] Wang R An J Ji F Jiao H Sun H Zhou D. Hypermethylation of the Keap1 gene in human lung cancer cell lines and lung cancer tissues. Biochem Biophys Res Commun (2008) 373:151–4. 10.1016/j.bbrc.2008.06.004 18555005

[45] Muscarella LA Parrella P D’Alessandro V la Torre A Barbano R Fontana A Frequent epigenetics inactivation of KEAP1 gene in non-small cell lung cancer. Epigenetics (2011) 6:710–9. 10.4161/epi.6.6.15773 21610322

[46] Eades G Yang M Yao Y Zhang Y Zhou Q. miR-200a regulates Nrf2 activation by targeting Keap1 mRNA in breast cancer cells. J Biol Chem (2011) 286:40725–33. 10.1074/jbc.M111.275495 21926171 PMC3220489

[47] Yamamoto S Inoue J Kawano T Kozaki K Omura K Inazawa J. The impact of miRNA-based molecular diagnostics and treatment of NRF2-stabilized tumors. Mol Cancer Res (2014) 12:58–68. 10.1158/1541-7786.MCR-13-0246-T 24307696

[48] Copple IM Lister A Obeng AD Kitteringham NR Jenkins RE Layfield R Physical and functional interaction of sequestosome 1 with Keap1 regulates the Keap1-Nrf2 cell defense pathway. J Biol Chem (2010) 285:16782–8. 10.1074/jbc.M109.096545 20378532 PMC2878012

[49] Jain A Lamark T Sjottem E Larsen KB Awuh JA Overvatn A p62/SQSTM1 is a target gene for transcription factor NRF2 and creates a positive feedback loop by inducing antioxidant response element-driven gene transcription. J Biol Chem (2010) 285:22576–91. 10.1074/jbc.M110.118976 20452972 PMC2903417

[50] Komatsu M Kurokawa H Waguri S Taguchi K Kobayashi A Ichimura Y The selective autophagy substrate p62 activates the stress responsive transcription factor Nrf2 through inactivation of Keap1. Nat Cell Biol (2010) 12:213–23. 10.1038/ncb2021 20173742

[51] Lau A Wang XJ Zhao F Villeneuve NF Wu T Jiang T A noncanonical mechanism of Nrf2 activation by autophagy deficiency: direct interaction between Keap1 and p62. Mol Cell Biol (2010) 30:3275–85. 10.1128/MCB.00248-10 20421418 PMC2897585

[52] Taguchi K Fujikawa N Komatsu M Ishii T Unno M Akaike T Keap1 degradation by autophagy for the maintenance of redox homeostasis. Proc Natl Acad Sci U S A (2012) 109:13561–6. 10.1073/pnas.1121572109 22872865 PMC3427110

[53] Ichimura Y Waguri S Sou YS Kageyama S Hasegawa J Ishimura R Phosphorylation of p62 activates the Keap1-Nrf2 pathway during selective autophagy. Mol Cell (2013) 51:618–31. 10.1016/j.molcel.2013.08.003 24011591

[54] Chen W Sun Z Wang XJ Jiang T Huang Z Fang D Direct interaction between Nrf2 and p21(Cip1/WAF1) upregulates the Nrf2-mediated antioxidant response. Mol Cell (2009) 34:663–73. 10.1016/j.molcel.2009.04.029 19560419 PMC2714804

[55] Gorrini C Baniasadi PS Harris IS Silvester J Inoue S Snow B BRCA1 interacts with Nrf2 to regulate antioxidant signaling and cell survival. J Exp Med (2013) 210:1529–44. 10.1084/jem.20121337 23857982 PMC3727320

[56] Adam J Hatipoglu E O’Flaherty L Ternette N Sahgal N Lockstone H Renal cyst formation in Fh1-deficient mice is independent of the Hif/Phd pathway: roles for fumarate in KEAP1 succination and Nrf2 signaling. Cancer Cell (2011) 20:524–37. 10.1016/j.ccr.2011.09.006 22014577 PMC3202623

[57] Ooi A Wong JC Petillo D Roossien D Perrier-Trudova V Whitten D An antioxidant response phenotype shared between hereditary and sporadic type 2 papillary renal cell carcinoma. Cancer Cell (2011) 20:511–23. 10.1016/j.ccr.2011.08.024 22014576

[58] Zhang Y Tang L. Discovery and development of sulforaphane as a cancer chemopreventive phytochemical. Acta Pharmacol Sin (2007) 28:1343–54. 10.1111/j.1745-7254.2007.00679.x 17723168

[59] Kwak MK Kensler TW. Targeting NRF2 signaling for cancer chemoprevention. Toxicol Appl Pharmacol (2010) 244:66–76. 10.1016/j.taap.2009.08.028 19732782 PMC3584341

[60] Leinonen HM Kansanen E Polonen P Heinaniemi M Levonen AL. Role of the Keap1-Nrf2 pathway in cancer. Adv Cancer Res (2014) 122:281–320. 10.1016/B978-0-12-420117-0.00008-6 24974185

[61] Lee S Lim MJ Kim MH Yu CH Yun YS Ahn J An effective strategy for increasing the radiosensitivity of human lung cancer cells by blocking Nrf2-dependent antioxidant responses. Free Radic Biol Med (2012) 53:807–16. 10.1016/j.freeradbiomed.2012.05.038 22684019

[62] Ren D Villeneuve NF Jiang T Wu T Lau A Toppin HA Brusatol enhances the efficacy of chemotherapy by inhibiting the Nrf2-mediated defense mechanism. Proc Natl Acad Sci U S A (2011) 108:1433–8. 10.1073/pnas.1014275108 21205897 PMC3029730

[63] Lee YR Yuan WC Ho HC Chen CH Shih HM Chen RH. The Cullin 3 substrate adaptor KLHL20 mediates DAPK ubiquitination to control interferon responses. EMBO J (2010) 29:1748–61. 10.1038/emboj.2010.62 20389280 PMC2876967

[64] Bialik S Kimchi A. DAP-kinase as a target for drug design in cancer and diseases associated with accelerated cell death. Semin Cancer Biol (2004) 14:283–94. 10.1016/j.semcancer.2004.04.008 15219621

[65] Bialik S Kimchi A. The DAP-kinase interactome. Apoptosis (2014) 19:316–28. 10.1007/s10495-013-0926-3 24220855

[66] Levin-Salomon V Bialik S Kimchi A. DAP-kinase and autophagy. Apoptosis (2014) 19:346–56. 10.1007/s10495-013-0918-3 24264886

[67] Chen HY Lee YR Chen RH. The functions and regulations of DAPK in cancer metastasis. Apoptosis (2014) 19:364–70. 10.1007/s10495-013-0923-6 24166138

[68] Ivanovska J Mahadevan V Schneider-Stock R. DAPK and cytoskeleton-associated functions. Apoptosis (2014) 19:329–38. 10.1007/s10495-013-0916-5 24166137

[69] Chen HY Lin YM Chung HC Lang YD Lin CJ Huang J miR-103/107 promote metastasis of colorectal cancer by targeting the metastasis suppressors DAPK and KLF4. Cancer Res (2012) 72:3631–41. 10.1158/0008-5472.CAN-12-0667 22593189

[70] Raval A Tanner SM Byrd JC Angerman EB Perko JD Chen SS Downregulation of death-associated protein kinase 1 (DAPK1) in chronic lymphocytic leukemia. Cell (2007) 129:879–90. 10.1016/j.cell.2007.03.043 17540169 PMC4647864

[71] Wang WJ Kuo JC Ku W Lee YR Lin FC Chang YL The tumor suppressor DAPK is reciprocally regulated by tyrosine kinase Src and phosphatase LAR. Mol Cell (2007) 27:701–16. 10.1016/j.molcel.2007.06.037 17803936

[72] Deiss LP Feinstein E Berissi H Cohen O Kimchi A. Identification of a novel serine/threonine kinase and a novel 15-kD protein as potential mediators of the gamma interferon-induced cell death. Genes Dev (1995) 9:15–30. 10.1101/gad.9.1.15 7828849

[73] Lavau C Marchio A Fagioli M Jansen J Falini B Lebon P The acute promyelocytic leukaemia-associated PML gene is induced by interferon. Oncogene (1995) 11:871–6. 7545807

[74] Stadler M Chelbi-Alix MK Koken MH Venturini L Lee C Saib A Transcriptional induction of the PML growth suppressor gene by interferons is mediated through an ISRE and a GAS element. Oncogene (1995) 11:2565–73. 8545113

[75] de The H Chomienne C Lanotte M Degos L Dejean A. The t(15;17) translocation of acute promyelocytic leukaemia fuses the retinoic acid receptor alpha gene to a novel transcribed locus. Nature (1990) 347:558–61. 10.1038/347558a0 2170850

[76] Bernardi R Guernah I Jin D Grisendi S Alimonti A Teruya-Feldstein J PML inhibits HIF-1alpha translation and neoangiogenesis through repression of mTOR. Nature (2006) 442:779–85. 10.1038/nature05029 16915281

[77] Bernardi R Pandolfi PP. Structure, dynamics and functions of promyelocytic leukaemia nuclear bodies. Nat Rev Mol Cell Biol (2007) 8:1006–16. 10.1038/nrm2277 17928811

[78] Reineke EL Liu Y Kao HY. Promyelocytic leukemia protein controls cell migration in response to hydrogen peroxide and insulin-like growth factor-1. J Biol Chem (2010) 285:9485–92. 10.1074/jbc.M109.063362 20100838 PMC2843199

[79] Salomoni P Ferguson BJ Wyllie AH Rich T. New insights into the role of PML in tumour suppression. Cell Res (2008) 18:622–40. 10.1038/cr.2008.58 18504460

[80] Carracedo A Weiss D Leliaert AK Bhasin M de Boer VC Laurent G A metabolic prosurvival role for PML in breast cancer. J Clin Invest (2012) 122:3088–100. 10.1172/JCI62129 22886304 PMC3433768

[81] Ito K Bernardi R Morotti A Matsuoka S Saglio G Ikeda Y PML targeting eradicates quiescent leukaemia-initiating cells. Nature (2008) 453:1072–8. 10.1038/nature07016 18469801 PMC2712082

[82] Gurrieri C Capodieci P Bernardi R Scaglioni PP Nafa K Rush LJ Loss of the tumor suppressor PML in human cancers of multiple histologic origins. J Natl Cancer Inst (2004) 96:269–79. 10.1093/jnci/djh043 14970276

[83] Chen RH Lee YR Yuan WC. The role of PML ubiquitination in human malignancies. J Biomed Sci (2012) 19:81. 10.1186/1423-0127-19-81 22935031 PMC3438505

[84] Lin YC Lu LT Chen HY Duan X Lin X Feng XH SCP phosphatases suppress renal cell carcinoma by stabilizing PML and inhibiting mTOR/HIF signaling. Cancer Res (2014) 74:6935–46. 10.1158/0008-5472.CAN-14-1330 25293974

[85] Wu HC Lin YC Liu CH Chung HC Wang YT Lin YW USP11 regulates PML stability to control Notch-induced malignancy in brain tumours. Nat Commun (2014) 5:3214. 10.1038/ncomms4214 24487962 PMC5645609

[86] Yuan WC Lee YR Huang SF Lin YM Chen TY Chung HC A Cullin3-KLHL20 ubiquitin ligase-dependent pathway targets PML to potentiate HIF-1 signaling and prostate cancer progression. Cancer Cell (2011) 20:214–28. 10.1016/j.ccr.2011.07.008 21840486

[87] Dellaire G Ching RW Dehghani H Ren Y Bazett-Jones DP. The number of PML nuclear bodies increases in early S phase by a fission mechanism. J Cell Sci (2006) 119:1026–33. 10.1242/jcs.02816 16492708

[88] Wenger RH Stiehl DP Camenisch G. Integration of oxygen signaling at the consensus HRE. Sci STKE (2005) 2005:re12. 10.1126/stke.3062005re12 16234508

[89] Semenza GL. Hypoxia-inducible factors: mediators of cancer progression and targets for cancer therapy. Trends Pharmacol Sci (2012) 33:207–14. 10.1016/j.tips.2012.01.005 22398146 PMC3437546

[90] Semenza GL. Defining the role of hypoxia-inducible factor 1 in cancer biology and therapeutics. Oncogene (2010) 29:625–34. 10.1038/onc.2009.441 19946328 PMC2969168

[91] Kan Z Jaiswal BS Stinson J Janakiraman V Bhatt D Stern HM Diverse somatic mutation patterns and pathway alterations in human cancers. Nature (2010) 466:869–73. 10.1038/nature09208 20668451

[92] Baca SC Prandi D Lawrence MS Mosquera JM Romanel A Drier Y Punctuated evolution of prostate cancer genomes. Cell (2013) 153:666–77. 10.1016/j.cell.2013.03.021 23622249 PMC3690918

[93] Barbieri CE Baca SC Lawrence MS Demichelis F Blattner M Theurillat JP Exome sequencing identifies recurrent SPOP, FOXA1 and MED12 mutations in prostate cancer. Nat Genet (2012) 44:685–9. 10.1038/ng.2279 22610119 PMC3673022

[94] Berger MF Lawrence MS Demichelis F Drier Y Cibulskis K Sivachenko AY The genomic complexity of primary human prostate cancer. Nature (2011) 470:214–20. 10.1038/nature09744 21307934 PMC3075885

[95] Grasso CS Wu YM Robinson DR Cao X Dhanasekaran SM Khan AP The mutational landscape of lethal castration-resistant prostate cancer. Nature (2012) 487:239–43. 10.1038/nature11125 22722839 PMC3396711

[96] An J Ren S Murphy SJ Dalangood S Chang C Pang X Truncated ERG oncoproteins from TMPRSS2-ERG fusions are resistant to SPOP-mediated proteasome degradation. Mol Cell (2015) 59:904–16. 10.1016/j.molcel.2015.07.025 26344096

[97] An J Wang C Deng Y Yu L Huang H. Destruction of full-length androgen receptor by wild-type SPOP, but not prostate-cancer-associated mutants. Cell Rep (2014) 6:657–69. 10.1016/j.celrep.2014.01.013 24508459 PMC4361392

[98] Gan W Dai X Lunardi A Li Z Inuzuka H Liu P SPOP promotes ubiquitination and degradation of the ERG oncoprotein to suppress prostate cancer progression. Mol Cell (2015) 59:917–30. 10.1016/j.molcel.2015.07.026 26344095 PMC4575912

[99] Li C Ao J Fu J Lee DF Xu J Lonard D Tumor-suppressor role for the SPOP ubiquitin ligase in signal-dependent proteolysis of the oncogenic co-activator SRC-3/AIB1. Oncogene (2011) 30:4350–64. 10.1038/onc.2011.151 21577200 PMC3158261

[100] Theurillat JP Udeshi ND Errington WJ Svinkina T Baca SC Pop M Prostate cancer. Ubiquitylome analysis identifies dysregulation of effector substrates in SPOP-mutant prostate cancer. Science (2014) 346:85–9. 10.1126/science.1250255 25278611 PMC4257137

[101] Zhu H Ren S Bitler BG Aird KM Tu Z Skordalakes E SPOP E3 ubiquitin ligase adaptor promotes cellular senescence by degrading the SENP7 deSUMOylase. Cell Rep (2015) 13:1183–93. 10.1016/j.celrep.2015.09.083 26527005 PMC4644472

[102] Chen CD Welsbie DS Tran C Baek SH Chen R Vessella R Molecular determinants of resistance to antiandrogen therapy. Nat Med (2004) 10:33–9. 10.1038/nm972 14702632

[103] Xu J Wu RC O’Malley BW. Normal and cancer-related functions of the p160 steroid receptor co-activator (SRC) family. Nat Rev Cancer (2009) 9:615–30. 10.1038/nrc2695 19701241 PMC2908510

[104] Zhou XE Suino-Powell KM Li J He Y Mackeigan JP Melcher K Identification of SRC3/AIB1 as a preferred coactivator for hormone-activated androgen receptor. J Biol Chem (2010) 285:9161–71. 10.1074/jbc.M109.085779 20086010 PMC2838335

[105] Geng C He B Xu L Barbieri CE Eedunuri VK Chew SA Prostate cancer-associated mutations in speckle-type POZ protein (SPOP) regulate steroid receptor coactivator 3 protein turnover. Proc Natl Acad Sci U S A (2013) 110:6997–7002. 10.1073/pnas.1304502110 23559371 PMC3637757

[106] Kumar-Sinha C Tomlins SA Chinnaiyan AM Recurrent gene fusions in prostate cancer. Nat Rev Cancer (2008) 8:497–511. 10.1038/nrc2402 18563191 PMC2711688

[107] Tomlins SA Rhodes DR Perner S Dhanasekaran SM Mehra R Sun XW Recurrent fusion of TMPRSS2 and ETS transcription factor genes in prostate cancer. Science (2005) 310:644–8. 10.1126/science.1117679 16254181

[108] Rosen P Sesterhenn IA Brassell SA McLeod DG Srivastava S Dobi A Clinical potential of the ERG oncoprotein in prostate cancer. Nat Rev Urol (2012) 9:131–7. 10.1038/nrurol.2012.10 22331093

[109] Le Gallo M O’Hara AJ Rudd ML Urick ME Hansen NF O’Neil NJ Exome sequencing of serous endometrial tumors identifies recurrent somatic mutations in chromatin-remodeling and ubiquitin ligase complex genes. Nat Genet (2012) 44:1310–5. 10.1038/ng.2455 23104009 PMC3515204

[110] Zhang P Gao K Jin X Ma J Peng J Wumaier R Endometrial cancer-associated mutants of SPOP are defective in regulating estrogen receptor-alpha protein turnover. Cell Death Dis (2015) 6:e1687 10.1038/cddis.2015.47 25766326 PMC4385925

[111] Gao K Jin X Tang Y Ma J Peng J Yu L Tumor suppressor SPOP mediates the proteasomal degradation of progesterone receptors (PRs) in breast cancer cells. Am J Cancer Res (2015) 5:3210–20. 26693071 PMC4656742

[112] Liu J Ghanim M Xue L Brown CD Iossifov I Angeletti C Analysis of *Drosophila* segmentation network identifies a JNK pathway factor overexpressed in kidney cancer. Science (2009) 323:1218–22. 10.1126/science.1157669 19164706 PMC2756524

[113] Gossage L Eisen T. Alterations in VHL as potential biomarkers in renal-cell carcinoma. Nat Rev Clin Oncol (2010) 7:277–88. 10.1038/nrclinonc.2010.42 20368728

[114] Li G Ci W Karmakar S Chen K Dhar R Fan Z SPOP promotes tumorigenesis by acting as a key regulatory hub in kidney cancer. Cancer Cell (2014) 25:455–68. 10.1016/j.ccr.2014.02.007 24656772 PMC4443692

[115] Kim B Nam HJ Pyo KE Jang MJ Kim IS Kim D Breast cancer metastasis suppressor 1 (BRMS1) is destabilized by the Cul3-SPOP E3 ubiquitin ligase complex. Biochem Biophys Res Commun (2011) 415:720–6. 10.1016/j.bbrc.2011.10.154 22085717

[116] Liu CC Lin YC Chen YH Chen CM Pang LY Chen HA Cul3-KLHL20 ubiquitin ligase governs the turnover of ULK1 and VPS34 complexes to control autophagy termination. Mol Cell (2016) 61:84–97. 10.1016/j.molcel.2015.11.001 26687681

